# 

**Published:** 1865-01

**Authors:** 


### CONTENTS.

### ORIGINAL CONTRIBUTIONS.

ART. I. An Article on Malignant Diphtheria, with Report of Eleven Cases. By D. W. Young, M.D., of Geneva, Ill., ............ 1
ART. II. Recent Advances in the Science of Light. By J. S. Jewell, M.D., Prof. Anatomy in Chicago Medical College, .......... 25
ART. III. Double Pneumonia, with Chronic Bronchitis, Cured by Sugar of Lead and Sulphate of Quinine. By G. C. Paoli, M.D., of Chicago, Ill. Read before the Chicago Medical Society, ............ 36

### SELECTIONS.

On the Therapeutic Value of Alkaline and Earthy Sulphites in the Treatment of Catalytic Diseases. By Dr. H. R. De Ricci, .................... 38
Letter from Dr. W. N. Cote, ........................................ 44
Treatment of Acute Orchitis by Puncturing the Testicle, .................. 47
Effects of the Excssive Use of Sugar on the System, ...................... 48
On the Condition of the Stomach and Intestines in Scarlatina, ............... 50

### BOOK NOTICES.

A Treatise on Military Surgery and Hygiene. By Frank Hastings Hamilton, M.D., ........................................ 56
Braithwaite's Retrospect of Practical Medicine and Surgery. Part I. Jan. 1865, ........................................ 56
London Lancet, ........................................ 56

### EDITORIAL.

Mortality of Chicago, for January, 1865, .................................. 56
Chicago Medical College Commencement, .................................. 56
Transactions of the American Medical Association for 1864, ............... 57
Illinois State Medical Society, .................................. 57
American Medical Association, .................................. 58
Arsenic-Eating in Styria, .................................. 58
Treatment of Onychia, .................................. 60
Respiration after Decapitation, .................................. 61
Treatment of Asthma, .................................. 61
Regeneration of Bone, .................................. 62
Obituary, .................................. 62

